# Pre- and intraoperative acoustic and functional assessment of the novel APrevent^®^ VOIS implant during routine medialization thyroplasty

**DOI:** 10.1007/s00405-019-05756-3

**Published:** 2019-12-16

**Authors:** Guan-Yuh Ho, Matthias Leonhard, Doris-Maria Denk-Linnert, Berit Schneider-Stickler

**Affiliations:** Division of Phoniatrics-Logopedics, Department of Otorhinolaryngology, Medical University of Vienna, Waehringer Guertel 18-20, 1090 Vienna, Austria

**Keywords:** Medialization thyroplasty, Vocal fold paresis/paralysis, Laryngology, UVFP, VOIS, TVFMI

## Abstract

**Purpose:**

Persistent unilateral vocal fold paralysis (UFVP) with glottal insufficiency often requires type I medialization thyroplasty (MT). Previous implants cannot be adjusted postoperatively if necessary. The newly developed APrevent^®^ VOIS implant (VOIS) can provide postoperative re-adjustment to avoid revision MT. The objective of this pilot study is to evaluate the VOIS intraoperatively concerning voice improvement, surgical feasibility and device handling.

**Methods:**

During routine MT, VOIS was applied short time in eight patients before the regular implantation of the Titanium Vocal Fold Medialization Implant (TVFMI™). In all patients, perceptual voice sound analysis using R(oughness)–B(reathiness)–H(oarseness)-scale, measurement of M(aximum)–P(honation)–T(ime) and glottal closure in videolaryngoscopy were performed before and after implanting VOIS/TVFMI™. Acoustic analyses of voice recordings were performed using freeware praat. Surgical feasibility, operative handling and device fitting of VOIS and TVFMI™ were assessed by the surgeon using V(isual)-A(nalog)-S(cale). Data were statistically analyzed with paired *t* test.

**Result:**

All patients showed significant improvement of voice sound parameters after VOIS/TVFMI™ implantation. The mean RBH-scale improved from preoperative *R* = 2.1, *B* = 2.3, *H* = 2.5 to *R* = 0.6, *B* = 0.3, *H* = 0.8 after VOIS and *R* = 0.5, *B* = 0.3, *H* = 0.8 after TVFMI™ implantation. The mean MPT increased from preoperative 7.9 to 14.6 s after VOIS and 13.8 s after TVFMI™ implantation. VOIS/TVFMI™ achieved complete glottal closure in 7/8 patients. The satisfaction with intraoperative device fitting and device handling of VOIS was as good as that of TVFMI™.

**Conclusion:**

The novel APrevent^®^ VOIS implant showed similar intraoperative voice improvement compared to routinely used TVFMI™ without adverse device events and with safe device fitting.

## Introduction

Unilateral vocal fold paralysis (UVFP) commonly occurs in patients with damage to the vagal nerve, its nucleus in the brainstem or its peripheral branch, the recurrent laryngeal nerve (RLN). It may be caused by stroke, head and neck injuries, tumors, neurological or infectious diseases, iatrogenic damage due to surgical trauma or idiopathic reasons.

Opening of the vocal folds is achieved by the activity of the M. cricoarytaenoideus posterior, and closing by activity of M. cricoarytaenoideus lateralis, M. interarytaenoideus and M. thyroarytaenoideus. All intrinsic laryngeal muscles are innervated by the RLN. In patients with UVFP, the ailing vocal fold cannot be opened and closed in unison with the contralateral vocal fold. The UVFP may lead to dysphonia, swallowing impairment with aspiration and breathing difficulties due to incomplete glottal closure [31].

Conventional surgical techniques to treat UVFP include type I medialization thyroplasty (MT) procedure with/without arytenoid rotation/adduction (AA) procedure [25]. Since the introduction of MT by Isshiki et al. [14] in 1974, MT has been widely accepted and performed as the standard phonosurgical procedure in patients with glottic insufficiency, in particular due to UVFP.

Isshiki [15] first described the AA procedure in 1978. It is an arytenoid rotation technique to move the vocal process medially, posteriorly and inferiorly, and thus also medializes the vocal fold. Since then, different methods of AA or arytenoid rotation techniques have been proposed [11, 28, 42, 45]. All of them are accomplished by tracking either directly on the muscular process of the arytenoid cartilage or on the intrinsic muscles inserting on the vocal process, namely thyroarytenoid muscle (TA) and lateral cricoarytenoid muscle (LCA) [43], mimicking the physiologic actions of the larynx during phonation. It is generally accepted that the disadvantage of AA is its inability to tense the anterior membranous vocal fold. Therefore, AA procedure is often performed in combination with MT procedure [3, 17, 22, 29].

Several studies have revealed that MT combined with AA resulted both in improvement of voice quality and prevented aspiration [2] and/or aspiration pneumonia [8, 25]. The major disadvantage is long surgical time and possible complications. Furthermore, the implant size and/or tension of sutures/thread used in the existing MT and AA procedures are not able to be precisely adjusted intra- and postoperatively. Often, it is difficult to accurately perform intraoperative adjustment of implant due to edematous swelling of laryngeal mucosa or surrounding soft tissues, caused by the procedure itself. Also, in case of atrophy of the affected laryngeal muscles, results cannot be optimized due to lack of postoperative adjustment [1].

Since the introduction of MT, various implant materials have been used, such as the silastic block [19, 20, 33, 44], pre-molded silastic [6, 16, 23, 26, 27, 30] from Montgomery^®^ and hydroxyapatite implants [8, 40, 41] from VoCOM^®^, polytetrafluoroethylene strips [10, 21, 22, 24, 30, 37, 39] from Gore-Tex^®^ and the titanium medialization implant [7, 9, 34, 36, 44], TVFMI™, from Heinz Kurz Medical GmbH.

All of them are permanent implants that achieve medialization of the impaired vocal fold through an adjacent thyroid cartilage window. The primary limitations of MT procedure include the inability to close a wide posterior glottal gap.

The existing implants are summarized below:

- Montgomery Thyroplasty Implant System (Boston Medical Products Inc.; FDA approved: K972317; CE approved)
- Titanium Vocal Fold Medialization Implant (TVFMI™) (Heinz Kurz Medical GmbH; FDA approval: K991324; CE approved)
- ThyroProtip (Alcis; CE approved)
- Vocal Cord Medialization System (VoCOM) (Gyrus ACMI Inc.; FDA approved: K974311)
- Gore Revox (W.L. Gore & Associates; FDA approved: K983525)

It was reported that revision rate of type I MT ranges from 8 to 33% [18]. The most common reasons for revision surgery involved replacement with a larger implant (37%; undercorrection), a smaller implant (24%; overcorrection), added vocal fold augmentation with injectables (19.7%; persistent glottic gap) and arytenoid adduction (10.3%; persistent posterior gap), not including cases with initial good voice result but subsequent long-term poor voice quality secondary to presumed vocal fold atrophy associated with the vocal fold paralysis, lasting 1–2 years starting from denervation [5, 32].

In 2011, Hoffman et al. introduced a concept of an adjustable balloon implant (ABI) for MT, demonstrating adequate and effective medialization with significant improvements in aerodynamic and acoustic parameters in an excised canine laryngeal model [12, 13].

**Fig. 1:**
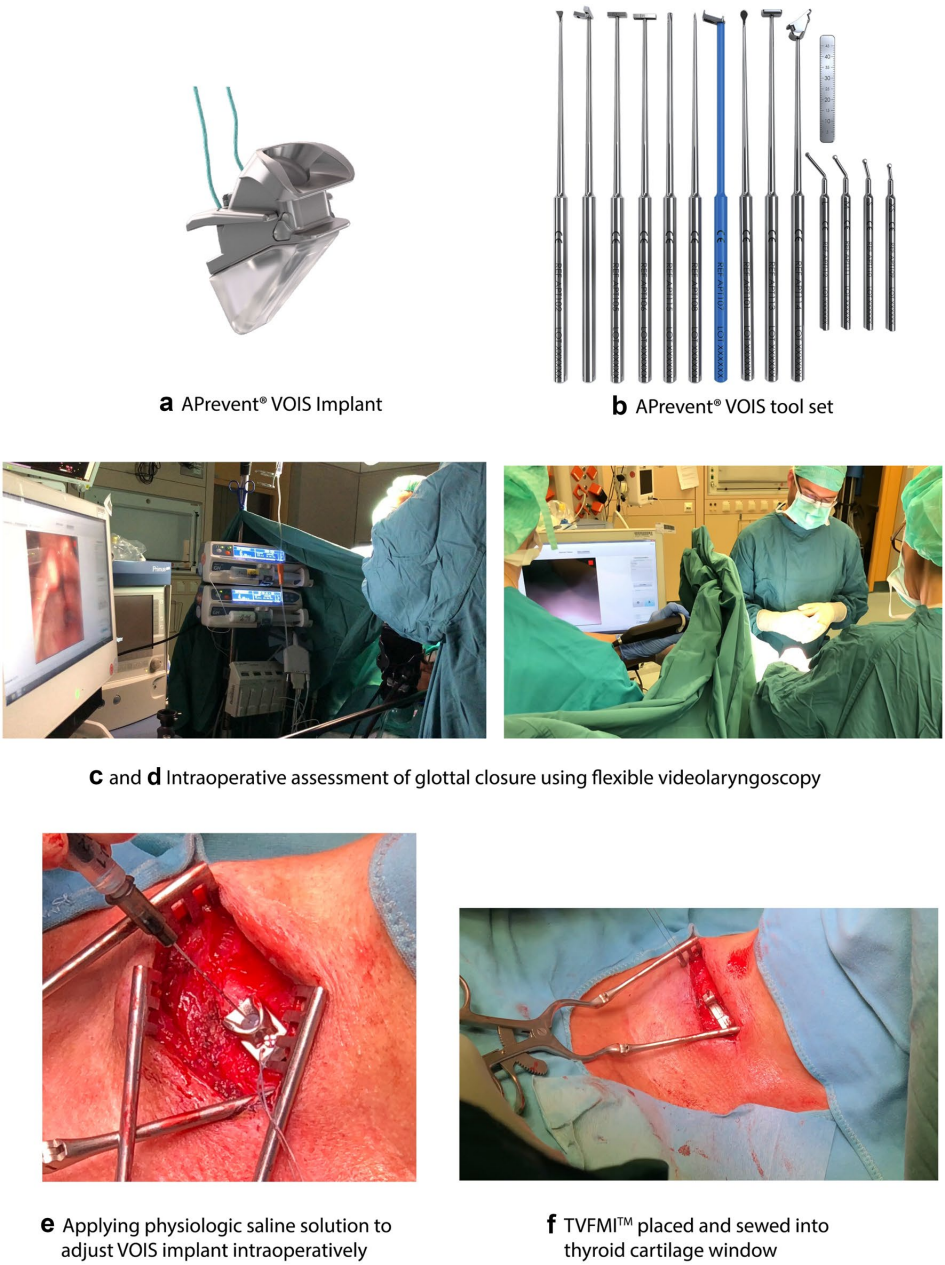
**a** APrevent^®^ VOIS implant, **b** APrevent^®^ VOIS tool set, **c**, **d** intraoperative assessment of glottal closure using flexible videolaryngoscopy, **e** applying physiologic saline solution to adjust VOIS implant intraoperatively, **f** TVFMI™ placed and sewed into thyroid cartilage window

In this pilot study, the novel APrevent^®^ VOIS implant (VOIS) (Fig. 1a) from APrevent Biotech GmbH based in Feldkirch Vorarlberg Austria was provided for its first use in patients with UVFP. The VOIS is made of inert materials, such as silicone and titanium, that have routinely been used for vocal fold medialization implants and long-term implants in other body parts (e.g., for urogenital or gastrointestinal organs) for decades. The surgical procedures are almost identical to the already existing procedures of MT. The VOIS is available in four sizes from x-small (XS) to large (L).

The VOIS can be adjusted and readjusted either intraoperatively or postoperatively if indicated, which may shorten the operation time and reduce the risk of perioperative complications and postoperative airway compromise. It provides the ability to achieve both vocal fold medialization and arytenoid adduction. The adjustable implant system can be customized for each individual’s need by varying the filling volumes both during and after the operation, thus enhancing optimization result for each patient.

Objectives of this pilot study were:

- to evaluate the voice quality before and after the temporary insertion of the VOIS during routine MT procedure with TVFMI™;
- to examine the perceptual voice sound scaling and acoustic variables prior and after implanting VOIS and TFVMI™;
- to assess the surgical feasibility and device fitting of both VOIS and TVFMI™ during the MT.

## Patients and methods

### Patients

This study has been approved by the Ethics Committee (EK) of the Medical University of Vienna with the EK number 1049/2018.

From July 2018 to February 2019, eight patients scheduled for routine MT using the TVFMI™ were included in this pilot study for testing the VOIS intraoperatively prior to the permanent implantation of the TVFMI™ implant. Of the eight patients, four were men with an average age of 53.5 years (age ranged from 42 to 59 years) and four were women with an average age of 55.5 years (age ranged from 29 to 79 years). All patients had an insufficient glottal closure caused by UVFP of different etiologies: status post-lung cancer (2 cases), status post-thyroidectomy (1 case), status post-carotid surgery (1 case), status post-esophagectomy (1 case), status post-paraganglioma extirpation (1 case), status post-cervical spine surgery (1 case) and laryngeal injury following intubation (1 case).

### Diagnostic and surgical procedures

Prior to the MT, the patients were comprehensively examined including anamnestic data, videolaryngostroboscopy, voice range profile measurements and voice function tests in order to indicate the best therapeutic options. All patients were comprehensively informed on the study purpose and the surgical procedure with the VOIS. After signing the consent form, the patients were enrolled in the study.

The surgical procedure was based on the techniques described by Friedrich [9]. The surgery was performed under local anesthesia combined with intravenous sedation (i.e., sedoanalgesia). The same surgeons performed the MT in all eight patients (BSS, ML).

**Fig. 2:**
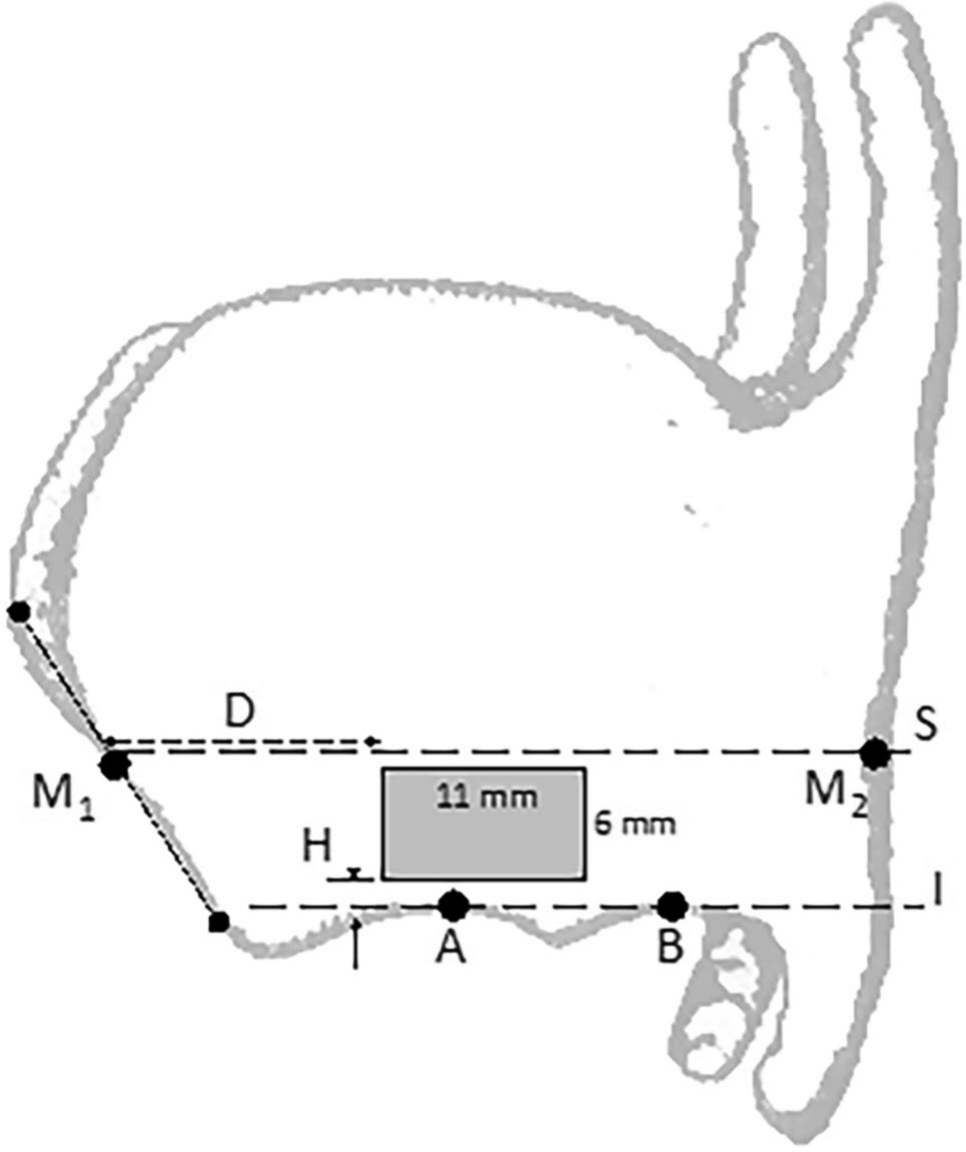
Key points for type I thyroplasty window. Point M1 is located on the midpoint of anterior thyroid cartilage border line, extending from incisura of the superior thyroid notch to the most anterior inferior edge of the thyroid cartilage. Point M2 is located on the posterior thyroid cartilage border. Point A and B are anterior and posterior to the inferior thyroid tubercle, respectively. The “Inferior Border Line I” passes through the points A and B. “Superior Line S” passing through points M1 and M2 is parallel to the “Inferior Border Line I” and corresponds to the horizontal level of the vocal folds and is used to evaluate the overall length of the thyroid cartilage (from anterior to posterior). Distance D along “Superior Line S,” an implant specific distance, describes the preliminary anterior margin of the thyroplasty window, chosen based on the overall length of the thyroid cartilage as shown in Fig. 2 or markers on the “Thyroid Cartilage Ruler”

After the patient has been sedated and given local anesthesia, skin incision, dissection and exposure of the thyroid cartilage were performed. After sufficient exposure of the ipsilateral thyroid cartilage lamina, under preservation of the perichondrium, key points (shown in Fig. 2) were allocated. The key points provide precise information of anatomical landmarks for the determination of the appropriate window location and size on the thyroid cartilage. For the allocation of the key points, specially designed tools and probe provided by the APrevent^®^ Biotech GmbH have been used (Fig. 1b): thyroid cartilage ruler, rectangular upper window edge caliper, linear anterior window edge caliper and cartilage marker.

After the allocation of the key points and with the aid of the provided instruments, the thyroplasty window making procedure could be performed easily. Following the cartilage fenestration, the VOIS with appropriate size was temporarily implanted. The MT was performed in three patients on the right vocal fold and in five patients on the left vocal fold. For the four male patients, one VOIS with size large and three with size medium were used. One VOIS with size small and three with size x-small were temporarily inserted into the female patients.

Before the temporal fixation of the VOIS, the air in the expandable silicone cushion was cleared by repeated and slowly flushing with a 0.9% physiologic saline solution in a 1-ml syringe (Fig. 1e). After flushing, the saline solution was partially removed to keep the implant in an unexpanded shape. For evaluation, the implant was placed into the cartilage window and temporally fixed to the thyroid cartilage. Following this, the silicone cushion was injected with 0.9% physiologic saline solution to adjust and optimize the vocal cord position. Following the intraoperative evaluation of the VOIS and its subsequent removal, the TVFMI™ (Fig. 1f) was placed into the cartilage window and sewn permanently to the thyroid (cartilage).

### Evaluation procedures

For determining the type of vocal fold closure according to the Södersten and Lindestad classification during phonation pre- and intraoperatively after temporary VOIS and permanent TVFMI™ implantation, examination using flexible videolaryngoscopy (Fig. 1c, d) was performed [38]. For this examination, the following devices were used: flexible videolaryngoscope and DiVAS Documentation System from Xion medical GmbH Berlin.

The functional voice assessment involved perceptual acoustic voice analysis. For the perceptual evaluation of sound, the RBH-scale commonly used in German-speaking countries was selected. The rating of parameters such as roughness (R), breathiness (B) and hoarseness (H) was done on a four-point scale; 0 = normal, 1 = mild deviance, 2 = moderate deviance and 3 = severe deviance. The subjective judgment and rating of the voice quality were based on conversational speech at habitual pitch and loudness. Further, patients were asked to sustain the vowels /a:/ , /i:/ and /u:/ at comfortable pitch and loudness for at least 2 s. The maximum phonation time (MPT) of sustained /a;/ following deep breath was also obtained from each patient. The functional voice assessment was performed preoperatively and after correct placement for both the VOIS and TVFMI™ implants.

For further acoustic analysis, digital voice recordings were also made pre- and intraoperatively. For the recordings a Sony IC Recorder, model ICD-SX2000 was applied. The distance from the microphone to the patient’s mouth was about 20 cm. For the semiquantitative acoustic data analysis, the freeware praat version 6.1 was used to determine the parameters: percentage jitter (jitter %), shimmer Decibel (shimmer dB), median pitch Hertz (f0 Hz) and harmonic-to-noise ratio Decibel (HNR dB) of the vowels /a:/, /i:/, and /u:/.

In addition, subjective surgeon’s satisfaction with the surgical procedure and handling of the VOIS and TVFMI™ was evaluated, respectively, using a visual analog scale (VAS).

### Statistical analysis

Paired *t* test was used to compare intraoperative data between the VOIS and TVFMI™. *P* values lower than 0.05 were of significance.

## Results

### Videolaryngoscopy

The flexible videolaryngoscopy showed improvement in glottal closure during phonation in all patients during surgery after insertion of either VOIS or TVFMI™. The judging of the glottal closure using flexible videolaryngoscopy was done referring to the rating protocol by Södersten and Lindestad [38]. Preoperatively, six patients (75%) had incomplete closure all along the folds, and two patients (25%) had incomplete closure of the posterior two thirds of the folds. After intraoperative placement of VOIS, a complete closure could be achieved in seven patients (87.5%), whereas one patient (12.5%) showed an incomplete closure in the cartilaginous part. Also, after the placement of TVFMI™, complete closure could be shown in seven patients (87.5%), and triangular incomplete closure of the posterior thirds of the folds was observed in one patient (12.5%).

### *Functional voice assessment: perceptual voice assessment (RBH*-*scale) and maximum phonation time (MPT)*

Preoperatively, the voice of the patients showed moderate roughness (mean 2.1), moderate breathiness (mean 2.3) and almost severe hoarseness (mean 2.5). With the VOIS temporally in place, all patients presented an improved intraoperative voice quality with almost no roughness (mean 0.6), no breathiness (mean 0.3) and only mild hoarseness (mean 0.8). With the implantation of TVFMI™, the voice quality of the patients showed similar results: almost no roughness (mean 0.5), no breathiness (mean 0.3) and at least mild degree of hoarseness (mean 0.8). The statistical analysis using paired *t* test showed *p* values lower than 0.05 for both the VOIS and TVFMI™, which means a significant improvement of the voice quality was achieved. Table 1 shows the RBH-scale prior and after VOIS/TVFMI™ implantation.

**Table 1:** Mean ± SD of RBH-scale and maximum phonation time (MPT)

	Preoperative	With VOIS	With TVFMI	*p* value		
				Comparison of pre- and intraoperative with VOIS	Comparison of pre- and intraoperative with TVFMI™	Comparison of intraoperative with VOIS and TVFMI™
Roughness R	2.1 ± 0.8	0.6 ± 0.7	0.5 ± 0.8	0.001	0.000	0.351
Breathiness B	2.3 ± 0.9	0.3 ± 0.5	0.3 ± 0.5	0.000	0.000	NA*
Hoarseness H	2.5 ± 0.8	0.8 ± 0.7	0.8 ± 0.7	0.000	0.000	NA*
MPT (s)	7.9 ± 3.0	14.7 ± 6.8	13.8 ± 6.4	0.014	0.007	0.645

*SD *standard deviation

*t cannot be computed because the standard error of the difference is 0

When assessing the MPT before and after correct placement of VOIS and TVFMI™, respectively, it also revealed major improvement in all patients. Whereas the MPT has an average of 7.9 s preoperatively, it could be increased to an average of 14.6 s after VOIS implant placement and 13.8 s after TVFMI™ implantation, respectively. In paired *t* test, the *p* values were lower than 0.05 for both VOIS implant and TVFMI™. The MPT before and after VOIS and TVFMI™ implantation is shown in Table 1.

### Acoustic voice analysis

Importing the voice files recorded preoperatively and with implantation of the VOIS and TVFMI™ to praat version 6.1, the parameters F0 Hz, jitter %, shimmer dB and HNR dB were calculated. The results are given in Table 2. Preoperatively, the average of mean jitter % was 2.2 ± 2.3. The average jitter % was 0.5 ± 0.2 after insertion of the VOIS and 0.6 ± 0.3 after TVFMI™ implantation. The average value of mean shimmer dB was 1.0 ± 0.5 preoperatively and 0.4 ± 0.2 after VOIS placement and 0.5 ± 0.3 after TVFMI™ implantation, respectively. The average of mean HNR dB improved from 11.4 ± 6.7 preoperatively to 17.6 ± 4.5 with VOIS and 17.7 ± 5.0 with TVFMI™. All findings revealed improvement after the implantation of both VOIS and TVFMI™.

**Table 2:** Mean statistics ± SD for F0, jitter, shimmer and HNR preoperative, with VOIS and TVFMI™

Acoustic parameters	Preoperative	With VOIS	With TVFMI	*p* value
				Comparison of intraoperative with VOIS and TVFMI™
Mean fundamental frequency F0 (Hz)	170.5 ± 59.8	177.3 ± 72.7	161.1 ± 14.5	0.492
Mean jitter (%)	2.2 ± 2.3	0.5 ± 0.2	0.6 ± 0.3	0.686
Mean shimmer (dB)	1.0 ± 0.5	0.4 ± 0.2	0.5 ± 0.3	0.887
Mean harmonic-to-noise ratio (dB)	11.4 ± 6.7	17.6 ± 4.5	17.7 ± 5.0	0.895
Total patients *n*	8	8	8	

*SD *standard deviation

When compared VOIS and TVFMI™ using paired *t* test regarding the results for acoustic data analysis (F0 Hz, jitter %, shimmer dB and HNR dB), no significant differences were found (*p* < 0.05).

### Subjective surgeon’s satisfaction (VAS index)

The subjective surgeon’s satisfaction regarding the surgical procedure and the VOIS/TVFMI™ using VAS is given in Table 3. It shows no differences between the two implants.

**Table 3:** Subjective surgeon’s satisfaction (VAS index with maximum = 10) ± SD

VAS scale	With VOIS	With TVFMI™	*P* value
Device fitting	9.8 ± 0.5	9.6 ± 0.7	0.612
Device handling	9.7 ± 0.5	9.9 ± 0.2	0.227
Overall satisfaction	9.9 ± 0.2	9.9 ± 0.2	NA*

*SD *standard deviation

*t cannot be computed because the standard error of the difference is 0

### Adverse events/adverse device effects

No adverse events occurred during the MT procedures using VOIS and TVFMI™ in all patients. Intraoperatively, no adverse device effects were observed when implanting the VOIS and TVFMI™ in each of the eight patients.

## Discussion

UVFP usually results in glottal insufficiency with dysphonia, dyspnea and swallowing impairment due to denervation of the ailing intrinsic laryngeal muscles. Glottic insufficiency causes insufficient transduction of aerodynamic energy into acoustic energy and leads to a rough, breathy and hoarse voice [4]. Within the laryngeal framework procedures, MT is a widely used surgical technique for treatment of unilateral vocal fold paralysis (UVFP).

In this study, for the first time, the novel APrevent^®^ medialization implant VOIS has been introduced, which should enable the surgeon to perform postoperative implant adjustment if necessary, by transcutaneous balloon refilling. In the first step, the efficacy of voice improvement, the surgical feasibility and the device handling of the VOIS were assessed in comparison with routinely used TVFMI™. For the evaluation, eight patients with permanent UVFP and insufficient glottal closure of various etiologies were enrolled and treated by vocal fold medialization using both implants—the newly developed VOIS temporarily and the routine TVFMI™ permanently. Prior to this study, between 2016 and 2018, the VOIS concept has been assessed by animal studies, which already confirmed the effectiveness and safety of the VOIS. Further, the VOIS was also tested in an excised larynx model and in fresh human laryngeal cadaver studies prior to this study. From 2017 to 2018, certain material tests have been applied to the VOIS to warrant its safety regarding the incorporated silicone cushion.

The VOIS has been designed in four sizes (x-small, small, medium and large). In this study, the sizes x-small and small have been used in women and, the sizes medium and large in men.

The preoperative degree of roughness, breathiness and hoarseness could be improved after implantation of VOIS as well as of TVFMI™. After MT, the voice quality of three out of eight patients as by RBH-scale returned to almost normal, whereas either only mild degrees of roughness and hoarseness or breathiness and hoarseness have been seen in the remaining five patients. The result of the voice quality by RBH-scale is comparable to previous study conducted by Schneider et al. in 2003 [35]. Concerning the glottal closure insufficiency, both implants revealed equally good results after insertion during MT. The acoustic parameter, maximum phonation time, has been significantly improved in all patients, too.

Although one of the most important methods to perceptually evaluate abnormal voice quality is using the RBH-scale by clinically well-trained listeners, its reliability is ambiguous due to intra-rater and inter-rater variabilities. Therefore, for clinical as well as for research purpose, an objective assessment of voice quality is of great importance [35]. For this study, the freeware praat, version 6.1 was used to analyze the acoustic data semiquantitatively. In all patients, the acoustic data analysis showed improvement in the parameters F0 Hz, jitter %, shimmer dB and HNR dB after VOIS as well as after TVFMI™ implantation. By comparing the results of acoustic data analysis of VOIS and TVFMI™ using paired *t* test, no significant differences were found (*p* < 0.05).

In this pilot study, no severe adverse events such as airway obstruction, severe tissue swelling or severe bleeding were observed. No displacement of the implant during filling procedure was detected. After VOIS was securely fixated into the thyroid cartilage window intraoperatively, no extrusion of implants was seen during phonation, coughing or throat clearing. The stability of the implant was also tested by pulling on the sliding sutures in lateral direction before and after the fixation plate was secured by a screw. None of the VOIS showed signs of dislocation from the designated position. In general, no severe adverse events and adverse device events were observed intraoperatively in all patients during the pilot study.

Like TVFMI™, the new VOIS ensures a precise and atraumatic medialization of the paralyzed vocal fold with excellent postoperative functional results. The instruments specifically designed for localization and creation of the window in the thyroid cartilage ala helped the surgeon to properly define the window position with reliable precision. Even though the surgeon accountable for this study has not participated in the development of the VOIS, following the perfectly described guidelines in the surgical protocol, the surgeon was able to choose the right implant size, to precisely position and fixate the implant into the thyroplasty window and to adjust the implant during surgery without any difficulties.

Further, for the VOIS, compared to other existing thyroplasty implants, an adequate medialization can be easily evaluated by injecting or removing 0.9% physiologic saline solution into/from the silicone cushion, without repeated implant insertion or removal. The advantage of using 0.9% physiologic saline solution is that it has the lowest diffusion rate through the silicone cushion. Thus, in the long term, it can ensure a constant filling volume of the silicone cushion.

In addition, the subjective surgeon’s satisfaction was also evaluated with a VAS scale, regarding device fitting, device handling and satisfaction of the operator. The VOIS is a newly developed system, including surgical instruments and implants for MT. Despite the noticeable slight difference in device-handling satisfaction between VOIS and TVFMI™ at the beginning of this study, in general, there were almost no differences noted in the VAS index between the VOIS and TVFMI™.

## Conclusion

The VOIS is made of inert materials, such as silicone and titanium, that have already been used for vocal fold medialization implants and long-term implants in other body parts (e.g., for urogenital or gastrointestinal organs) for decades. Its implantation follows the routine surgical procedure of MT. In this study, comparable satisfying results regarding voice improvement due to better glottal closure, improved acoustic voice parameters and improved perceptual voice quality could be shown for VOIS in comparison with the routinely used TVFMI™.

In this pilot study, VOIS implants could achieve comparable intraoperative voice improvements to those of TVFMI™. Since VOIS offers the additional benefit of intra- and postoperative fine volume adjustments, it is therefore of great interest to further evaluate functional results of its permanent implantation in the following planned long-term study.
